# Enhanced anti-PD-1 therapy in hepatocellular carcinoma by tumor vascular disruption and normalization dependent on combretastatin A4 nanoparticles and DC101

**DOI:** 10.7150/thno.58164

**Published:** 2021-04-03

**Authors:** Xin Bao, Na Shen, Yan Lou, Haiyang Yu, Yue Wang, Linlin Liu, Zhaohui Tang, Xuesi Chen

**Affiliations:** 1 Department of Radiotherapy, The Second Hospital of Jilin University, Changchun 130041, P. R. China.; 2 Key Laboratory of Polymer Ecomaterials, Changchun Institute of Applied Chemistry, Chinese Academy of Sciences, Changchun 130022, P. R. China.; 3 Department of Thyroid, The Second Hospital of Jilin University, Changchun, 130041, P. R. China.; 4 Department of Nephropathy, The Second Hospital of Jilin University, Changchun, 130041, P. R. China.

**Keywords:** combretastatin A4, nanoparticles, DC101, anti-PD-1 antibody, hepatocellular carcinoma

## Abstract

Anti-programmed cell death protein 1 (PD-1) therapy has shown promising efficacy in hepatocellular carcinoma (HCC), but its response rates in advanced HCC are lower than 20%. A critical reason for this is the imbalance between CD8^+^ T cells and tumor burden. Here, a novel concept of vascular disruption and normalization dependent on a polymeric vascular disrupting agent (VDA) poly (_L_-glutamic acid)-*graft*-methoxy poly (ethylene glycol)/combretastatin A4 (CA4-NPs) + a vascular endothelial growth factor (VEGF)/VEGF receptor 2 (VEGFR2) inhibitor DC101 is applied to improve anti-PD-1 therapy, wherein CA4-NPs reduce tumor burden and DC101 simultaneously increases the number of intratumoral CD8^+^ T cells, successfully regulating the abovementioned imbalance in an H22 tumor model.

**Methods:** Blood vessel density, tumor cell proliferation, and necrosis were evaluated to reveal the effects on reducing tumor burden by CA4-NP treatment. Pericyte coverage of blood vessels, tumor blood vessel perfusion, tumor hypoxia, and intratumoral immune cells were examined to verify their role in vascular normalization and immune cell homing of DC101. Furthermore, the effects of CA4-NPs + DC101 on reducing tumor burden and increasing the number of immune cells were studied. Finally, tumor suppression, intratumoral CD8^+^ T cell activation, and the synergistic effects of anti-PD-1 combined with CA4-NPs + DC101 were verified.

**Results:** The tumor inhibition rate of anti-PD-1 antibody combined with CA4-NPs + DC101 reached 86.4%, which was significantly higher than that of anti-PD-1 (16.8%) alone. Importantly, the Q value reflecting the synergy between CA4-NPs + DC101 and anti-PD-1 was 1.24, demonstrating a strong synergistic effect. Furthermore, CA4-NPs + DC101 improved anti-PD-1 therapy by increasing the number of intratumoral CD8^+^ T cells (anti-PD-1, 0.31% vs triple drug combination, 1.18%).

**Conclusion:** These results reveal a novel approach to enhance anti-PD-1 therapy with VDAs + VEGF/VEGFR2 inhibitors in HCC.

## Introduction

Hepatocellular carcinoma (HCC) is the fourth leading cause of cancer-related death worldwide 1. As early HCC is asymptomatic and disease progression is rapid, majority of patients are diagnosed at an advanced stage, eliminating the possibility of surgical resection or transplantation. Clinically, the therapeutic strategies for advanced HCC are limited, mainly including transarterial chemoembolization (TACE) and oral sorafenib therapy. TACE is a locoregional therapeutic approach blocking the principal tumor-feeding arteries for local disease control 2. Because TACE induces hypoxia and upregulates vascular endothelial growth factor (VEGF) and fibroblast growth factor (FGF), the incidence of recurrence is high 3. The oral multikinase inhibitor sorafenib was FDA-approved as a first-line treatment for advanced HCC in 2007 4. However, sorafenib only extends the overall survival of HCC patients by approximately 3 months and is associated with significant adverse effects 5, 6.

More recently, checkpoint blockade immunotherapy targeting programmed cell death protein 1 (PD-1) has produced promising clinical efficacy in HCC patients. Nivolumab and pembrolizumab, two humanized monoclonal antibodies targeting PD-1, were granted accelerated approval by the FDA for advanced HCC treatment as a second-line treatment following phase II clinical trials 7, 8. Unfortunately, the clinical efficacy of anti-PD-1 antibody (anti-PD-1) as a monotherapy is limited with an objective response rate (ORR) of 20% or less 9. Considering the unprecedented rates of long-lasting anti-tumor responses following anti-PD-1 treatment 10, methods to enhance its therapeutic efficacy are urgently required.

The immune checkpoint molecule PD-1 is well-known to bind to its ligand programmed cell death ligand 1 (PD-L1), leading to T cell exhaustion 11, 12. Thus, antibodies targeting PD-1 can prevent the PD-1-PD-L1 interaction, alleviating the inhibitory effect of PD-1 on T cells, including CD8^+^ T cells 13. The number of CD8^+^ T cells is used to predict the response to anti-PD-1 therapy 14, but this remains suboptimal in a clinical setting. Emerging evidence indicates that the failure of anti-PD-1 in cancer treatment partly results from the imbalance between CD8^+^ T cells and tumor burden, and the therapeutic efficacy of anti-PD-1 is positively associated with the ratio of CD8^+^ T cell invigoration to the tumor burden (measured as the sum of the long axis of all lesions, cm) 15. When the number of CD8^+^ T cells is comparable between small and large tumors, CD8^+^ T cells in the larger tumors may not be sufficient to effectively inhibit tumor growth. Methods to reduce the tumor burden and simultaneously increase the number of intratumoral CD8^+^ T cells can therefore enhance the therapeutic efficacy of anti-PD-1 for the treatment of HCC.

As HCC has a hyper-vascular structure 16, vascular disrupting agents (VDAs) can reduce HCC tumor burden by selectively disrupting established tumor blood vessels 17, 18. Furthermore, as TACE is a local treatment method that cannot completely block the feeding arteries of patients with multifocal HCC 19, systemic administration of VDAs is an alternative strategy to reduce the tumor burden. Combretastatin A4 (CA4) is a representative VDA 20. CA4 disodium phosphate (CA4P), a phosphate prodrug of CA4, has entered phase III clinical trials 21. However, compared with CA4P, CA4 nanoparticles (CA4-NPs) display enhanced tumor blood vessel targeting and tumor inhibition because of the low permeability of nanoparticles in tumor blood vessels 22. Thus, CA4-NPs can reduce the tumor burden more effectively.

Although the use of CA4-NPs can reduce the tumor burden *in vivo*, it frequently also results in high VEGF expression. VEGF is a tumor derived pro-angiogenic factor that stimulates angiogenesis 23. Emerging evidence suggests that the ectopic overexpression of VEGF results in a highly abnormal vasculature 24. Abnormal tumor blood vessels prevent the infiltration of immune effector cells, including CD8^+^ T cells, into the tumor, impairing the therapeutic efficacy of anti-PD-1 25-27. Recent studies have suggested that blocking VEGF/VEGF receptor 2 (VERFR2) signaling can transiently normalize the tumor vasculature and increase the number of intratumoral CD8^+^ T cells 28-32. In clinical practice, bevacizumab, a humanized anti-VEGF monoclonal antibody, fails to improve survival when used as a monotherapy, but increases survival when used in combination with chemotherapy or immunotherapy 33-37. A potential explanation for these findings is that bevacizumab “normalizes” the tumor vasculature, improving the delivery of anticancer drugs and increasing the number of intratumoral CD8^+^ T cells 38-40. VEGF/VEGFR2 inhibitors potentially block the function of VEGF in response to CA4-NPs, transiently normalizing the tumor vasculature, and increasing the number of intratumoral CD8^+^ T cells. Accordingly, the combination of CA4-NPs and VEGF/VEGFR2 inhibitors has the potential to reduce tumor burden, simultaneously increasing the number of intratumoral CD8^+^ T cells.

In this study, we proposed the concept of vascular disruption and normalization dependent on a polymeric VDA (poly (_L_-glutamic acid)-*graft*-methoxy poly (ethylene glycol)/combretastatin A4, CA4-NPs) + a VEGF/VEGFR2 inhibitor DC101 for improving anti-PD-1 therapy, and investigated the effectiveness and mechanism of CA4-NPs + DC101 in enhancing anti-PD-1 therapy in an H22 tumor model. Blood vessel density, tumor cell proliferation, and necrosis were evaluated to reveal the effects of CA4-NP treatment on reducing tumor burden. Pericyte coverage of blood vessels, tumor blood vessel perfusion, tumor hypoxia, and intratumoral immune cells were also examined to reveal their roles in vascular normalization and the immune cell homing of DC101. Furthermore, the effects of CA4-NPs + DC101 on reducing tumor burden and increasing the number of intratumoral immune cells were studied. Finally, the effects such as tumor suppression and intratumoral CD8^+^ T cell activation and synergistic effects of anti-PD-1 combined with CA4-NPs + DC101 were verified. Here, we revealed the ability of CA4-NPs + DC101-based combination therapy to enhance the therapeutic efficacy of anti-PD-1 in HCC.

## Methods

### Materials

**Figure 1:**
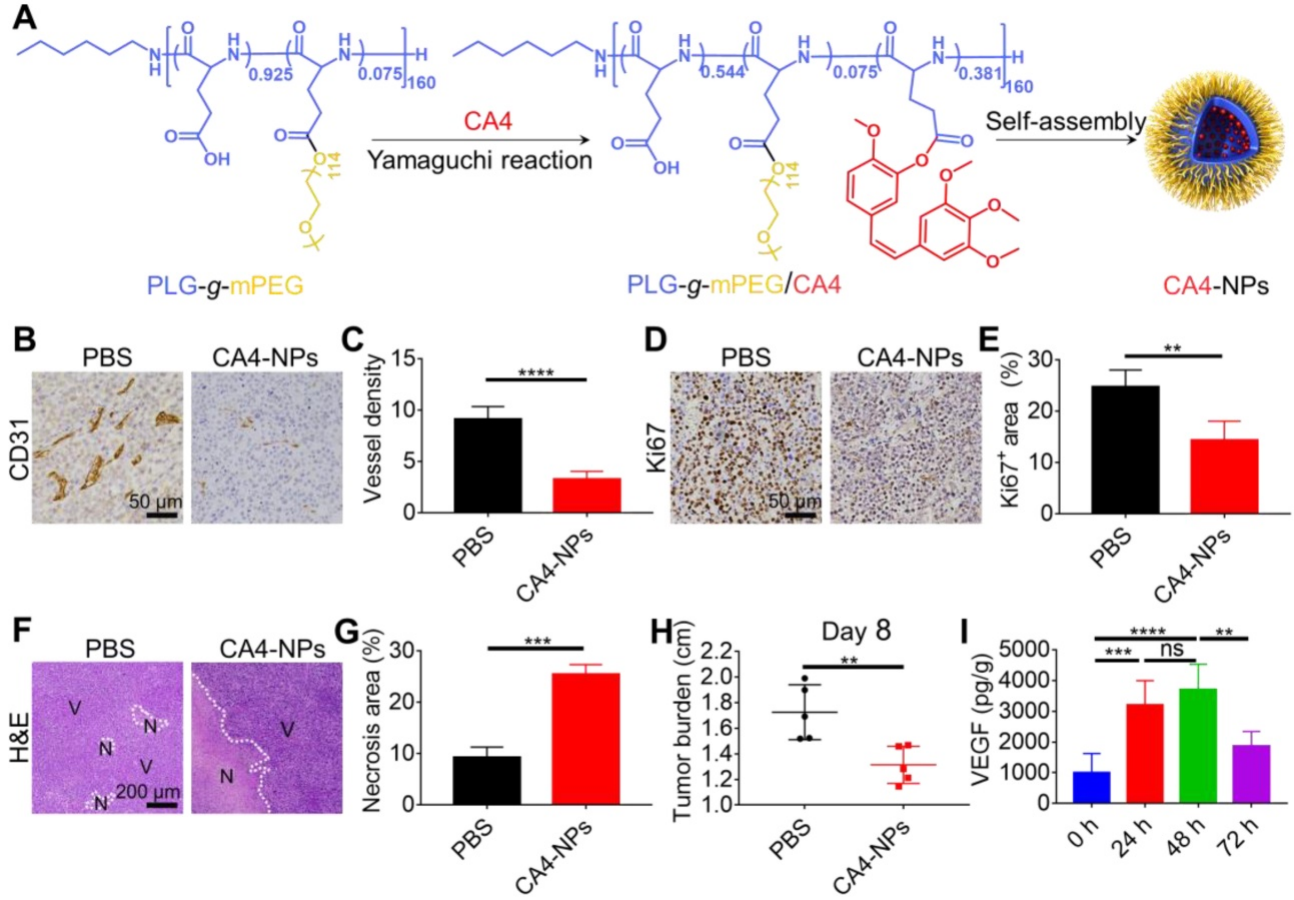
** CA4-NPs reduced the tumor burden.** H22 cells were subcutaneously inoculated into Balb/c mice. When the tumor volume reached ~170 mm^3^, CA4-NPs were intravenously administered at 45 mg/kg (on a CA4 basis) on day 0. (A) Schematic image showing the preparation of CA4-NPs. (B) IHC staining of CD31 in the tumors on day 2 after CA4-NP treatment. (C) Quantitative analysis of the microvessel density (MVD, which is represented as No. per field) for the IHC staining of CD31 in tumors. Seven high-density vascular areas (“hot spots”) of tumors were observed using an optical microscope at 100×, and then CD31^+^ vessels were photographed and counted at 400× in each respective hot spot. (D) IHC staining of Ki67 in the tumors on day 2 after CA4-NP treatment. (E) Quantitative analysis of the Ki67^+^ area (Ki67^+^ area/field) in IHC staining using Image J software (n = 5). (F) H&E staining of tumors in the PBS and CA4-NP groups on day 2. V, viable region of tumors; N, necrotic region of tumors. (G) Quantitative analysis of the necrotic area (necrotic area/field) for H&E staining of the tumors using Image J software (n = 3). (H) Tumor burden (long axis of tumors) in the PBS and CA4-NP groups on day 8 (n = 5). (I) ELISA for evaluating the intratumoral VEGF level at 0 h, 24 h, 48 h, and 72 h post-CA4-NP treatment (n = 5). Data are presented as mean ± SD (**P < 0.01, ***P < 0.001, ****P < 0.0001, ns, not significant).

CA4-NPs were synthesized via the Yamaguchi esterification reaction as previously described (Figure 1A) 22. Briefly, PLG-*g*-mPEG (4.00 g), CA4 (1.00 g, 3.16 mmol), 2, 4, 6-trichlorobenzoyl chloride (1.56 g, 6.40 mmol), DMAP (0.46 g, 3.77 mmol), and triethylamine (TEA, 0.9 mL, 6.46 mmol) were mixed in N, N-Dimethylformamide (DMF) and incubated at 60 °C for 6 h. The reaction mixtures were then precipitated into excess diethyl ether, re-dissolved in DMF, and dialyzed in distilled water (MWCO 3500). CA4-NPs were obtained after lyophilization. High-performance liquid chromatography (HPLC) was performed to measure the drug loading content (DLC, wt%) of CA4. In brief, the CA4-NPs (10.0 mg) were dissolved in 10 mL Milli Q water, and 0.4 mL 1 M NaOH was added, shaking at 37 °C for 1 h to produce free CA4. Next, 0.4 mL of 1.4 M phosphoric acid was added. HPLC (Waters 1525 system equipped with a reverse-phase column Symmetry^®^ C18) was used to determine the DLC of CA4 by monitoring at 305 nm. Elutions were performed with acetonitrile (or methanol) and water (v/v=4:1) pumped at a flow velocity of 1.0 mL/min at 25 °C. DLC was calculated by the following equation: DLC (%) = (amount of loaded drug/amount of nanoparticles) × 100, and the DLC of CA4-NPs was 15.9 wt%.

### Cell lines and animal models

H22 cells were purchased from the Beina Chuanglian Biotechnology Institute (Beijing, China). Female Balb/c mice (6-8 weeks old, 20 ± 2 g weight) were purchased from Vital River Laboratory Animal Technology Co., Ltd (Beijing, China). Female Kunming mice (6-8 weeks old, 20 ± 2 g weight) were provided by the Laboratory Animal Center of Jilin University (Changchun, China). All animal procedures were performed in accordance with the Guidelines for Care and Use of the Laboratory Animals approved by Jilin University and experiments were approved by the Animal Ethics Committee of Jilin University.

### H22 subcutaneous tumor models, *in vivo* antitumor effects, and tumor burden evaluation

H22 cells were injected into the abdomen of female Kunming mice (6-8 weeks old, 20 ± 2 g weight) under aseptic conditions for one week. H22 ascites were then transferred to the abdomen of other female Kunming mice (6-8 weeks old, 20 ± 2 g weight). One week later, ascites were suctioned and washed with phosphate-buffered saline (PBS; 0.01 M, pH 7.4) twice. The H22 subcutaneous tumor model was prepared by subcutaneously injecting 2 × 10^6^ H22 cells into the right flank of female Balb/c mice (6-8 weeks old, 20 ± 2 g weight). When the tumor volume reached ~170 mm^3^, mice were randomly divided into eight groups, including PBS (Group 1); CA4-NPs (Group 2); DC101 (Group 3); anti-PD-1 (Group 4); CA4-NPs + DC101 (Group 5); CA4-NPs + anti-PD-1 (Group 6); DC101 + anti-PD-1 (Group 7); and CA4-NPs + DC101 + anti-PD-1 (Group 8). The initiation of treatment was defined as day 0. CA4-NPs (45 mg/kg on a CA4 basis) were administered by intravenous (i.v.) injection via the tail vein on day 0. DC101 (BioXCell, 10 mg/kg) (Table S1) was administered by intraperitoneal (i.p.) injection on days 2, 5, and 8. Anti-PD-1 (BioXCell, 100 μg/mouse) (Table S1) was administered by i.p. injection on days 4, 7, and 10. Tumor volumes and body weights were recorded each day. The tumor volume (V) was calculated using the following equation: V = a × b^2^/2, where *a* represented the longest axis and *b* represented the shortest axis of the tumor. Tumor burden was defined as the tumoral long axis. The tumor growth inhibition rate (IR) was calculated using the following equation: IR (%) = [(TVc - TVt)/TVc] × 100, where *TVc* and *TVt* represented the mean tumor volumes in the control and treatment groups, respectively. The Q value was used to evaluate the synergistic effect between CA4-NPs + DC101 and anti-PD-1. The Q value was calculated using the following equation: Q = E_(A+B)_/[E_(A)_ + E_(B)_ - E_(A)_ × E_(B)_], where *E_(A+B)_*, *E_(A)_*, and *E_(B)_* represented the tumor inhibition rates of the combination group A + B, group A, and group B, respectively. Q < 0.85 indicated an antagonist effect of combination A with B. Further, 0.85 ≤ Q < 1.15 indicated an additive effect of combination A with B, and Q ≥ 1.15 indicated a synergistic effect of combination A with B 41. For survival analysis, when the tumor volume reached ~2000 mm^3^ or disease aggravation was observed, the mice were considered dead and survival time was recorded.

### Hematoxylin and eosin (H&E) staining

The procedure for H22 tumor modeling and drug treatment was the same as that followed for the tumor inhibition experiment. Tumor tissues in the PBS and CA4-NP groups were collected and subjected to H&E staining to verify tumor cell necrosis on day 2 (n = 3). Tumor tissues in the PBS, CA4-NP + DC101, anti-PD-1, and CA4-NP + DC101 + anti-PD-1 groups were collected and subjected to H&E staining to verify tumor cell necrosis on day 11 (n = 3). The major organs (heart, liver, spleen, lung, and kidney) of mice in the eight groups were collected and subjected to H&E staining to investigate systemic toxicity of drugs on day 11 (n = 3). The tumor tissues and the major organs were excised and fixed in 4% paraformaldehyde. Tissues were sectioned at a thickness of 5 µm and stained with H&E 42. Histological images were obtained using an optical microscope (Olympus, Japan).

### Immunohistochemical (IHC) staining

The procedure for H22 tumor modeling and drug treatment was the same as that followed for the tumor inhibition experiment. Tumor tissues in the PBS and CA4-NP groups were collected and subjected to IHC staining to examine the density of blood vessels and tumor cell proliferation on day 2 (n = 5). Tumor tissues in the PBS, CA4-NP + DC101, anti-PD-1, and CA4-NP + DC101 + anti-PD-1 groups were collected and subjected to IHC staining to examine tumor cell proliferation on day 11 (n = 3). Tumor tissues in the PBS, CA4-NP, DC101, CA4-NP + DC101, anti-PD-1, and CA4-NP + DC101 + anti-PD-1 groups were collected and subjected to IHC staining to examine the number of intratumoral CD8^+^ T cells on day 11. Tumor tissues were fixed in 4% paraformaldehyde, embedded in paraffin, and sectioned at a thickness of 5 μm. The slides were then heated in a dry oven at 60 °C for 30 min, deparaffinized in dimethylbenzene, rehydrated through a graded ethanol series, and rinsed in deionized water. Sections were then microwaved in 10 mM citric acid buffer (pH 6.0) at 70% power for 10 min for antigen retrieval. Endogenous peroxidase was quenched in 3% hydrogen peroxide for 20 min followed by rinsing with PBS 43. Non-specific binding was blocked with 10% goat serum at 37 °C for 40 min. Sections were then probed with anti-mouse platelet-endothelial cell adhesion molecule (CD31) (1:500) (Abcam), anti-mouse Ki67 antibodies (1:50) (Servicebio), or anti-CD8 rabbit antibody (1:500) (Servicebio) (Table S1) at 4 °C overnight, followed by labeling with peroxidase-conjugated secondary antibody (1:100) (Servicebio) (Table S1) at 37 °C for 1 h. After washing, the sections were stained with 3, 3'-diaminobenzidine (DAB) and counterstained with hematoxylin. Histological images were obtained using an optical microscope (Olympus, Japan). Tumor blood vessel counts were determined as per Weidner's method 44.

### Pericyte coverage evaluation

The procedure for H22 tumor modeling and drug treatment was the same as that followed for the tumor inhibition experiment. Tumor tissues of the PBS, DC101, and CA4-NP + DC101 groups were collected and subjected to pericyte coverage evaluation on day 10 (n = 3). Tumors were embedded in the optimal cutting temperature (OCT) compound. Frozen sections were blocked with 10% goat serum at 37 °C for 40 min. Sections were probed with APC-labeled rat anti-mouse CD31 antibody (1:100) (Thermo Fisher Scientific), and mouse anti-mouse α-smooth muscle actin (α-SMA) antibody (1:500) (Abcam) at 4 °C overnight, followed by labeling with Cy3-labeled goat anti-mouse secondary antibody (1:200) (ABclonal) at 37 °C for 1 h (Table S1). Nuclei were counterstained with 4′, 6-diamidino-2-phenylindole dihydrochloride (DAPI) (Table S2). Fluorescent images were obtained using a confocal laser-scanning microscope (Carl Zeiss, Germany).

### Small molecule Hoechst 33342 perfusion assays

The process for H22 tumor modeling and drug treatment was the same as that followed for the tumor inhibition experiment. Tumor tissues of the PBS, DC101, and CA4-NP + DC101 groups were collected and subjected to Hoechst 33342 perfusion assays on day 10 (n = 3). Mice were administered an i.v. injection of 40 mg/kg Hoechst 33342 (Dalian Meilun Biotech Co., Ltd). After 5 min, mice were anesthetized with isoflurane and fixed on an operation table. The skin of the chest was disinfected with complexing iodophors, and an incision was made in the middle of chest to expose the heart. The needle of a syringe containing 20 mL PBS was inserted into the left ventricle of the mouse. Simultaneously, the right auricular appendage was cut off with scissors. Subsequently, PBS was injected into the left ventricle for systemic perfusion. Tumors were removed and fixed in 4% paraformaldehyde for 2 h, and then soaked into 20% (w/v) and 30% (w/v) PBS-buffered sucrose solution successively at 4 °C until the tumors sank to the bottom of the tubes 45. The tumors were then embedded in the OCT compound and cut into 5-μm-thick sections. Nuclei were stained with SYTOX Green (Thermo Fisher) (Table S2). Images of the tumors were obtained using a confocal laser scanning microscope (Carl Zeiss, Germany).

### FITC-lectin perfusion assays

The process for H22 tumor modeling and drug treatment was the same as that followed for the tumor inhibition experiment. Tumor tissues of the PBS, DC101, and CA4-NP + DC101 groups were collected and subjected to FITC-lectin (FITC-labeled tomato lectin) perfusion assays on day 10 (n = 3). Mice were administered an i.v. injection of 10 mg/kg FITC-lectin (Sigma-Aldrich) in PBS (200 μL). After 10 min, tumors were excised, embedded in the OCT compound, and cut into 5-μm-thick sections. Slices were blocked with 10% goat serum at 37 °C for 40 min and incubated with APC-labeled anti-mouse CD31 antibody (1:100) (Thermo Fisher Scientific) at 4 °C overnight. Nuclei were stained with DAPI. Images of the tumors were obtained using a confocal laser scanning microscope (Carl Zeiss, Germany).

### Hypoxia assays

The procedure for H22 tumor modeling and drug treatment was the same as that followed for the tumor inhibition experiment. Tumor tissues of the PBS, DC101, and CA4-NP + DC101 groups were collected and subjected to hypoxia assays on day 10 (n = 3). Mice were administered an i.p. injection of 60 mg/kg pimonidazole hydrochloride (Hypoxyprobe, Inc) and sacrificed after 90 min. Tumor tissues were then collected, fixed in 4% paraformaldehyde for 2 h, and successively soaked in 20% (w/v) and 30% (w/v) PBS-buffered sucrose solution at 4 °C until the tumor tissues sank to the bottom of the tubes. Tissues were then embedded in the OCT compound and cut into 5-μm-thick sections. Frozen tissue sections were then blocked with 10% goat serum at 37 °C for 40 min, and incubated with Dylight™549 fluorophore-labeled anti-mouse IgG1 antibody (HP-Red549, 1:100) at 4 °C overnight. Nuclei were stained with DAPI. Images of the tumors were obtained using a confocal laser scanning microscope (Carl Zeiss, Germany).

### Enzyme-linked immunosorbent assay (ELISA)

The procedure for H22 tumor modeling and drug treatment was the same as that followed for the tumor inhibition experiment. To observe changes in intratumoral VEGF levels following CA4-NP treatment (45 mg/kg, on a CA4 basis), VEGF expression was assessed at 0, 24, 48, and 72 h (n = 5). Tumors were then excised and homogenized at a ratio of 1.0 g tissue/9.0 mL double distilled (dd) water and centrifuged at 3000 *g* for 20 min. Supernatants were then collected and assayed for VEGF level using a Mouse-VEGF ELISA kit (Lengton) according to the manufacturer's recommendation (Table S3). Tumor tissues of the PBS, CA4-NP + DC101, anti-PD-1, and CA4-NP + DC101 + anti-PD-1 groups were collected to analyze intratumoral interferon-gamma (IFN-γ) and tumor necrosis factor-alpha (TNF-α) levels on day 11 (n = 3) using mouse-IFNgamma (Anoric) and mouse-TNFalpha (Anoric) ELISA kits according to the manufacturer's recommendations, respectively (Table S3). Tumors were then excised and homogenized at a ratio of 1.0 *g* tissue/8.0 mL dd water, and centrifuged at 3000 *g* for 10 min. The obtained supernatants were then assayed for IFN-γ and TNF-α levels. Tumor tissues of the PBS, CA4-NP + DC101, anti-PD-1, and CA4-NP + DC101 + anti-PD-1 groups were collected to analyze intratumoral IL-2 levels on day 11 (n = 3) using IL-2 mouse uncoated ELISA Kit (Thermo Fisher) according to the manufacturer's recommendation (Table S3). The tumor tissues were homogenized at a ratio of 1.0 *g* tissue/10.0 mL dd water, and centrifuged at 3000 *g* for 10 min. The obtained supernatants were then assayed for IL-2 levels.

### Flow cytometry analysis

The procedure for H22 tumor modeling and drug treatment was the same as that followed for the tumor inhibition experiment. Tumor tissues of the PBS and DC101 groups were collected and analyzed by flow cytometry on day 10 (n = 3) to detect changes in T cell number caused by DC101-mediated vascular normalization. The tumor tissues of PBS, CA4-NP, and CA4-NP + DC101 groups were collected on day 10 (n = 3) and analyzed by flow cytometry to detect the changes of T cells caused by CA4-NP + DC101-mediated vascular normalization, simultaneously excluding the contribution of CA4-NPs. Tumor tissues of the PBS, CA4-NP, DC101, and CA4-NP + DC101 groups were collected and analyzed by flow cytometry on day 10 (n = 3) to detect the changes of intratumoral PD-L1 expression after treatment. Tumor tissues of the PBS, CA4-NP + DC101, anti-PD-1, and CA4-NP + DC101 + anti-PD-1 groups were collected to detect intratumoral T cells by flow cytometry to evaluate the synergistic effects of anti-PD-1 with CA4-NPs + DC101 on day 11 (n = 6). Tumors were sectioned into small pieces and digested using tumor dissociation buffer containing 0.8 mg/mL type IA collagenase, 0.1 mg/mL DNase I, and 0.1 mg/mL hyaluronidase at 37 °C for 1 h. Following digestion, tumor samples were homogenized by repeated pipetting and passed through a nylon filter in complete Roswell Park Memorial Institute (RPMI, Gibco) media supplemented with 10% fetal bovine serum (FBS). Single-cell suspensions were then incubated with the following fluorescent-labeled antibodies at 4 °C for 40 min: FITC-labeled anti-mouse CD3 (BioLegend), PE/Cy7-labeled anti-mouse CD4 (Invitrogen) or PE-labeled anti-mouse CD4 (Invitrogen), APC-labeled anti-mouse CD8 (BioLegend) antibodies, and PE-labeled anti-mouse PD-L1 (Table S1). Stained cells were then washed in FACS buffer, fixed in 4% paraformaldehyde, and subjected to flow cytometry. The total cells of tumor collected by the instrument were 100,000, which were the same among different groups. Data were analyzed using FlowJo V10 software.

### Statistical analysis

Data are expressed as the mean ± standard deviation (SD). Statistical differences for multiple groups were compared by one-way ANOVA. Individual groups were compared via Student's t-test. For survival analyses, log rank tests were performed for comparison. *P < 0.05 was considered statistically significant, **P < 0.01 was considered highly significant, ***P < 0.001 and ****P < 0.0001 were considered extremely significant, and ns was considered not significant.

## Results and Discussion

### CA4-NPs reduced tumor burden

CA4-NPs were prepared as shown in Figure 1A. Murine H22 cells were inoculated into syngeneic Balb/c mice to develop the HCC tumor model. Considering the role of CA4-NPs in disrupting tumor blood vessels, tumor hypoxia, and necrosis 46, the density of blood vessels, tumor cell proliferation, and necrosis were examined after a single injection of CA4-NPs (45 mg/kg, on a CA4 basis). Endothelial cells of the tumor blood vessels were identified by IHC staining of CD31 on day 2 of treatment (Figure 1B, C). The results indicated that the number of tumor blood vessels per field in the CA4-NP group (3.3 ± 0.8) was significantly lower than that in the PBS group (9.1 ± 1.2), indicating that the CA4-NPs effectively blocked tumor blood vessels. As Ki67 expression positively correlates with cell proliferation 47, intratumoral Ki67 expression on day 2 of treatment was evaluated (Figure 1D). Quantitative analysis indicated that the Ki67^+^ area in the CA4-NP group (14.4 ± 3.7%) was significantly lower than that in the PBS group (24.7 ± 3.3%) (Figure 1E). The result indicated that CA4-NPs effectively inhibited tumor cell proliferation in H22 tumor-bearing mice. H&E staining was performed to verify tumor cell necrosis on day 2 of treatment. Nuclear fragmentation was observed in the necrotic area (Figure 1F), which significantly increased in the CA4-NP group compared with that in the PBS group (Figure 1G). As shown in Figure 1H, compared with the PBS group, the CA4-NP group showed a decrease in tumor burden (long axis of tumors) by ~23.8% on day 8. These results indicated that CA4-NPs reduced the HCC tumor burden effectively by disrupting tumor blood vessels, inhibiting tumor cell proliferation, and enhancing tumor cell necrosis.

### DC101 increased the number of intratumoral CD8^+^ T cells

Emerging evidence suggests that VDAs lead to high VEGF expression in tumors 48. To observe changes in VEGF levels following CA4-NP treatment (45 mg/kg, on a CA4 basis), VEGF expression was assessed at 0, 24, 48, and 72 h by ELISA, and peak expression was revealed on day 2 of CA4-NP therapy (989.1 ± 638.5 pg/g, 3195.1 ± 802.2 pg/g, 3697.0 ± 836.2 pg/g, and 1873.2 ± 470.5 pg/g, respectively, Figure 1I). Day 2 was therefore selected for the co-administration of the VEGF/VERFR2 inhibitor DC101. It is well-documented that low-dose DC101 (10 mg/kg) administered as four doses at 3-day intervals can normalize the tumor vasculature, increasing the number of intratumoral CD8^+^ T cells 45. Tumor vascular normalization induced by DC101 was observed following 2 days of treatment and lasted for up to 8 days 49. Three doses of DC101 (10 mg/kg) at 3-day intervals were therefore administered to achieve vascular normalization in the H22 tumor model.

**Figure 2:**
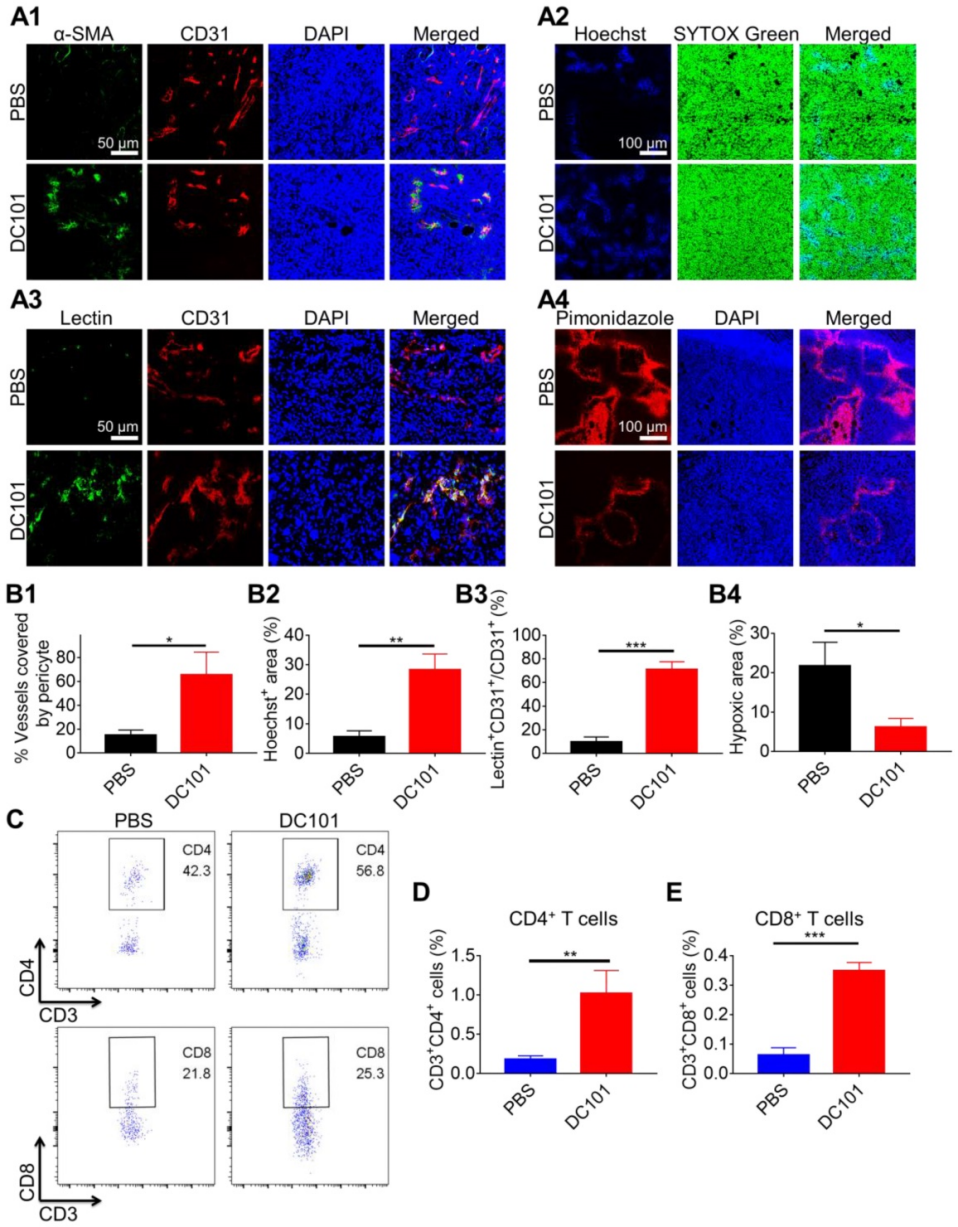
** DC101 normalized the tumor vasculature and increased the number of intratumoral CD8^+^ T cells.** H22 cells were subcutaneously inoculated into Balb/c mice. When the tumor volume reached ~170 mm^3^, DC101 10 mg/kg was administered through i.p. injection on days 2, 5, and 8. (A1) Immunofluorescent confocal images of the tumors showed blood vessel pericyte coverage in the PBS and DC101 groups on day 10. CD31^+^ endothelial cells, red; α-SMA^+^ pericytes, green; nuclei, blue. (A2) Immunofluorescent images for the Hoechst 33342 (Hoechst) tumor perfusion assay in the PBS and DC101 groups on day 10. Hoechst 33342^+^ cells, blue; nuclei, green. (A3) Immunofluorescent images of the tumors showing FITC-lectin (lectin) tumor blood vessel perfusion in the PBS and DC101 groups on day 10. CD31^+^ endothelial cells, red; FITC-lectin-perfused vessels, green; nuclei, blue. (A4) Immunofluorescent images showing the level of hypoxia in the PBS and DC101 groups on day 10. Pimonidazole^+^ cells, red; nuclei, blue. (B1) Quantification of pericyte coverage determined through α-SMA^+^CD31^+^/CD31^+^ staining using ZEN software (Carl Zeiss, Germany) (n = 3). (B2) Quantification of tumor perfusion determined through the Hoechst 33342^+^ area/field using the Image J software (n = 3). (B3) Quantification of perfused functional tumor blood vessels determined by lectin^+^CD31^+^/CD31^+^ staining using ZEN software (Carl Zeiss, Germany) (n = 3). (B4) Quantification of tumor hypoxia determined by pimonidazole^+^ area/tumor area using ZEN software (Carl Zeiss, Germany) (n = 3). (C) Representative flow cytometry plots for intratumoral CD4^+^ T (CD3^+^CD4^+^) cells and CD8^+^ T (CD3^+^CD8^+^) cells in the PBS and DC101 groups on day 10. (D, E) Quantification of intratumoral CD4^+^ T cells (D, n = 3) and CD8^+^ T cells (E, n = 3) detected by flow cytometry. Data are presented as mean ± SD (*P < 0.05, **P < 0.01, ***P < 0.001).

Tumor vasculature normalization in response to DC101 treatment was verified through the assessment of pericyte coverage 50, 51. We used pericyte marker α-SMA and endothelial cell marker CD31 to evaluate vascular structural normalization on day 10. As shown in Figure 2A1, colocalization of α-SMA (green) and CD31 (red) represented structurally normalized tumor vasculature. Pericyte coverage was 65.6 ± 19.1% in the DC101 group compared with 14.9 ± 4.4% in the PBS group, suggesting that DC101 treatment significantly increased pericyte coverage (Figure 2B1). Normalization of the vascular structure induced by DC101 treatment would be expected to be accompanied by functional changes. Therefore, we assessed vessel function by analyzing for tumor blood vessel perfusion and hypoxia. Hoechst 33342 is a low-molecular-weight DNA stain that is rapidly taken up by cells with limited diffusibility across cell layers 52, 53. Hoechst 33342 specifically labels the nuclei of endothelial cells of functionally perfused blood vessels and their adjacent cells, and can therefore be used as a fluorescent probe to evaluate functional blood vessel supply 54-56. As shown in Figure 2A2, Hoechst 33342^+^ staining (blue fluorescence) significantly increased on day 10 following DC101 treatment (28.3 ± 5.3%) (Figure 2B2) compared with that after PBS treatment (5.7 ± 2.0%), indicating that DC101 improved tumor perfusion. FITC-lectin binds to glyco-conjugates of endothelial cells and can be used to reflect perfused blood vessels 57, 58. As shown in Figure 2A3, the colocalization of FITC-lectin (green) and CD31 (red) represented perfused vessels. Compared with the PBS group (9.7 ± 4.4%), the DC101 group (71.1 ± 6.5%) showed significantly higher numbers of perfused blood vessels on day 10 (Figure 2B3). Further, pimonidazole is a hypoxic probe that can be used to assess tumor hypoxia 59. In Figure 2A4, the red fluorescence represents the tumor hypoxia area. Compared with the PBS group (21.8 ± 6.0%), the DC101 group (6.3 ± 2.2%) showed lower level of pimonidazole staining in tumor sections on day 10 (Figure 2B4), indicating that DC101 reduced tumor hypoxia. Previous studies have demonstrated that normalized tumor vasculature increased the infiltration of intratumoral T cells 60. As DC101 normalized the morphology and function of the HCC tumor vasculature, we analyzed the relative abundance of intratumoral T cells on day 10 following DC101 treatment. The number of intratumoral CD4^+^ T cells was 1.0% in the DC101 group and 0.2% in the PBS group (Figure 2C, D), indicating increased CD4^+^ T cell infiltration following DC101 treatment. The number of intratumoral CD8^+^ T cells in the DC101 group (0.3%) was 2.0-fold higher than that in the PBS group (0.1%) (Figure 2C, E). Tumors were also identified by IHC staining of CD8 on day 11 of DC101 treatment (Figure S1). The results indicated that the number of CD8^+^ cells in the DC101 group was higher than that in the PBS group, confirming that DC101 treatment resulted in an increase in the number of intratumoral CD8^+^ T cells. These observations suggested that low-dose DC101 significantly increased the number of intratumoral T cells, including CD8^+^ T cells. As shown in Figure S2, compared with the PBS group, the DC101 group showed no significant decrease in tumor burden (long axis of tumors) on day 8. That result indicated that low-dose DC101 had no significant effect on reducing tumor burden.

### CA4-NPs + DC101 reduced tumor burden while simultaneously increasing the number of intratumoral CD8^+^ T cells

**Figure 3:**
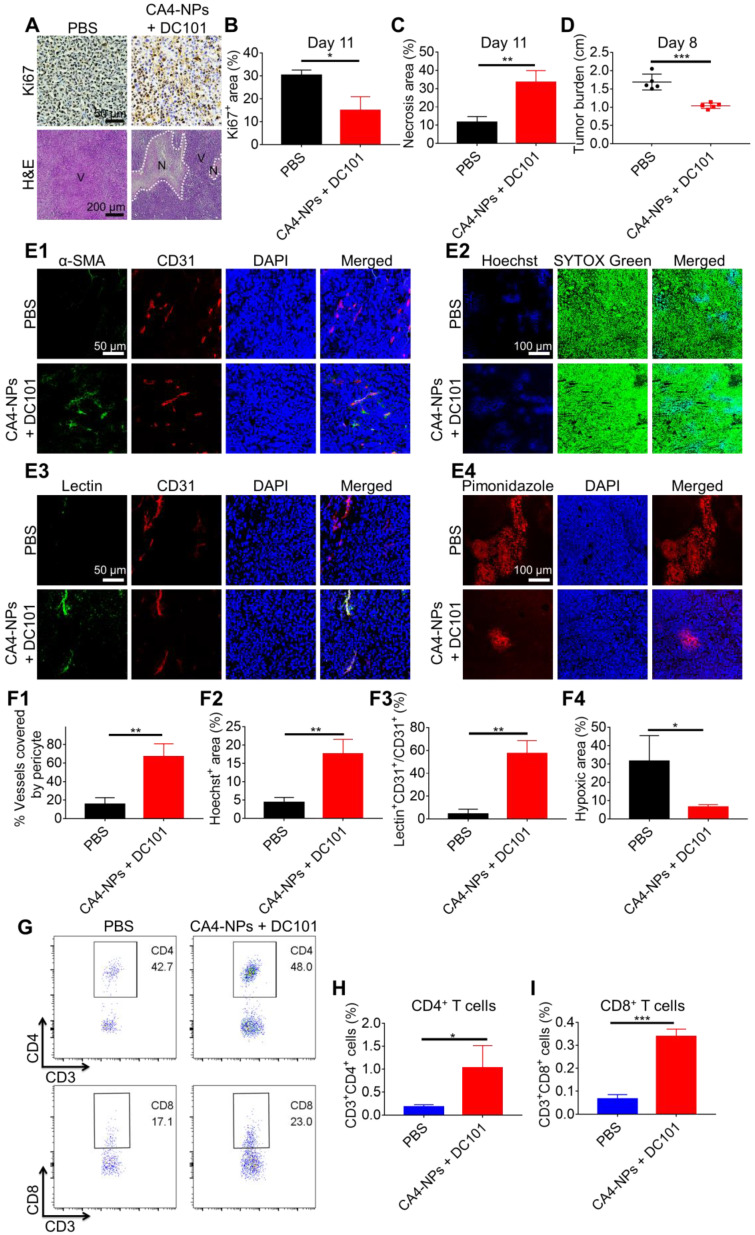
** CA4-NPs + DC101 reduced the tumor burden while simultaneously increasing the number of intratumoral CD8^+^ T cell**s**.** H22 cells were subcutaneously inoculated into Balb/c mice. When the tumor volume reached ~170 mm^3^, CA4-NPs (45 mg/kg, on a CA4 basis) were administered intravenously on day 0. DC101 (10 mg/kg) was administered intraperitoneally on days 2, 5, and 8. (A) Tumor IHC staining of Ki67 and H&E staining in the PBS and CA4-NP + DC101 groups on day 11. V, viable region of the tumors; N, necrotic region of the tumors. (B) Quantitative analysis of the Ki67^+^ area (Ki67^+^ area/field) for tumor IHC staining using Image J software (n = 3). (C) Quantitative analysis of the necrotic area (necrotic area/field) for tumor H&E staining using Image J software (n = 3). (D) Tumor burden (long axis of tumors) in the PBS and CA4-NP + DC101 groups on day 8 (n = 5). (E1) Immunofluorescent images of the tumors showing blood vessel pericyte coverage in the PBS and CA4-NP + DC101 groups on day 10. CD31^+^ endothelial cells, red; α-SMA^+^ pericytes, green; nuclei, blue. (E2) Immunofluorescent images of the tumors showing Hoechst 33342 tumor perfusion in the PBS and CA4-NP + DC101 groups on day 10. Hoechst 33342^+^ cells, blue; nuclei, green. (E3) Immunofluorescent images of tumors showing FITC-lectin tumor blood vessel perfusion in the PBS and CA4-NP + DC101 groups on day 10. CD31^+^ endothelial cells, red; FITC-lectin-perfused vessels, green; nuclei, blue. (E4) Immunofluorescent images of the tumors showing hypoxia in the PBS and CA4-NP + DC101 groups on day 10. Pimonidazole^+^ cells, red; nuclei, blue. (F1) Quantification of pericyte coverage determined by α-SMA^+^CD31^+^/CD31^+^ staining using ZEN software (Carl Zeiss, Germany) (n = 3). (F2) Quantification of tumor perfusion determined by Hoechst 33342^+^ area/field using Image J software (n = 3). (F3) Quantification of perfused functional tumor blood vessels determined by lectin^+^CD31^+^/CD31^+^ staining using ZEN software (Carl Zeiss, Germany) (n = 3). (F4) Quantification of tumor hypoxia determined by the pimonidazole^+^ area/tumor area using ZEN software (Carl Zeiss, Germany) (n = 3). (G) Representative flow cytometry plots for intratumoral CD4^+^ T (CD3^+^CD4^+^) cells and CD8^+^ T (CD3^+^CD8^+^) cells in the PBS and CA4-NP + DC101 groups on day 10. (H, I) Quantification of intratumoral CD4^+^ T cells (H, n = 3) and CD8^+^ T cells (I, n = 3) detected by flow cytometry. Data are presented as mean ± SD (*P < 0.05, **P < 0.01, ***P < 0.001).

The data obtained to this point verified that the CA4-NPs reduced tumor burden, whilst low-dose DC101 increased the number of intratumoral CD8^+^ T cells. We next investigated whether the combination of CA4-NPs and DC101 together could reduce the tumor burden and increase the number of intratumoral CD8^+^ T cells. The effects of CA4-NPs + DC101 on tumor inhibition were verified through Ki67 and H&E staining (Figure 3A). Quantitative analysis of Ki67 on day 11 of treatment showed that the Ki67^+^ area in the CA4-NP + DC101 and PBS groups were 15.0 ± 6.0% and 30.3 ± 2.3%, respectively (Figure 3B), indicating that the combination of CA4-NPs and DC101 significantly inhibited tumor cell proliferation. H&E staining showed that, compared with the PBS group (11.5 ± 3.2%), the CA4-NP + DC101 group (33.5 ± 6.4%) had a significantly larger area of tumor necrosis on day 11 (Figure 3C). These data suggested that tumor cell proliferation was inhibited and that the cells became necrotic following CA4-NP + DC101 treatment. As shown in Figure 3D, compared with the PBS group, the CA4-NP + DC101 group showed a decrease in tumor burden (long axis of tumors) by ~38.3% on day 8. These results demonstrated that CA4-NPs + DC101 markedly reduced tumor burden.

We next evaluated whether the combination of CA4-NPs and DC101 could normalize the tumor vasculature and increase the number of intratumoral CD8^+^ T cells. As shown in Figure 3E1, the colocalization of CD31 (red) and α-SMA (green) represented a fraction of CD31-positive endothelial vessels surrounded by α-SMA-positive pericytes. The pericyte coverage was 67.0 ± 13.9% in the CA4-NP + DC101 group compared with 15.5 ± 7.1% in the PBS group (Figure 3F1). These data indicated that CA4-NP + DC101 treatment led to tumor vascular normalization with improved pericyte coverage. Next, tumor perfusion was determined by Hoechst 33342 perfusion assay. The tumor functional blood vessel supply region showed blue fluorescence (Hoechst 33342) (Figure 3E2). The CA4-NP + DC101 group displayed an increased Hoechst 33342^+^ area on day 10 (CA4-NPs + DC101, 17.7 ± 3.9% vs PBS, 4.4 ± 1.4%, Figure 3F2). Another tumor blood vessel perfusion experiment was performed with FITC-lectin. As shown in Figure 3E3, the colocalization of FITC-lectin (green) and CD31 (red) represented perfused vessels. As FITC-lectin only binds to endothelial cells of perfused blood vessels, the percentage of FITC-lectin perfused blood vessels would be expected to increase following vasculature normalization. Compared with the PBS group (4.5 ± 4.1%), the CA4-NP + DC101 group (57.5 ± 11.1%) showed significantly increased functionally perfused vessels on day 10 (Figure 3F3). Next, tumor hypoxia was evaluated by pimonidazole staining, which was a hypoxia probe that can be used to assess tumor hypoxia. As shown in Figure 3E4, hypoxic region labeled with the red fluorescence was 6.5 ± 1.3% in the CA4-NP + DC101 group compared with 31.6 ± 13.9% in the PBS group (Figure 3F4), indicating that tumor hypoxia was significantly reduced on day 10 following CA4-NP + DC101 treatment. Collectively, these data revealed the ability of CA4-NP + DC101 co-treatment to normalize tumor vasculature, leading to increased tumor pericyte coverage, improved tumor blood vessel perfusion, and reduced tumor hypoxia. With the normalization of tumor vasculature, changes in the number of intratumoral T cells were evaluated on day 10 using flow cytometry (Figure 3G). The number of CD4^+^ T cells in the CA4-NP + DC101 group (1.0%) was 4.0-fold higher than that in the PBS group (0.2%) (Figure 3H, and Figure S3, 4). Compared with the PBS group (0.1%), the CA4-NP + DC101 group (0.3%) led to a 2.0-fold increase in the number of intratumoral CD8^+^ T cells (Figure 3I, and Figure S3, 4). IHC staining of CD8 confirmed that CA4-NP + DC101 treatment resulted in an increase the number of intratumoral CD8^+^ T cells (Figure S5). Furthermore, the increase in CD4^+^ and CD8^+^ T cell proportions was mediated by DC101, rather than CA4-NPs. Taken together, these results demonstrated that CA4-NP + DC101 treatment reduced tumor burden while simultaneously increasing the number of intratumoral CD8^+^ T cells. Since there is clinical evidence that response rates to PD-1-blocking antibodies are higher in patients with high PD-L1 expression 61, The PD-L1 level in tumors was checked on day 10 by flow cytometry after CA4-NP + DC101 treatment. Figure S6 shows that the number of PD-L1^+^ cells in the PBS, CA4-NP, DC101, and CA4-NP + DC101 groups were 2.7 ± 0.2%, 3.6 ± 0.6%, 3.5 ± 1.4%, 4.9 ± 2.6%, respectively. PD-L1 expression did not significantly increase after CA4-NP + DC101 treatment.

### The combination of CA4-NPs and DC101 enhanced the therapeutic efficacy of anti-PD-1 in HCC with tolerable systemic toxicity

**Figure 4:**
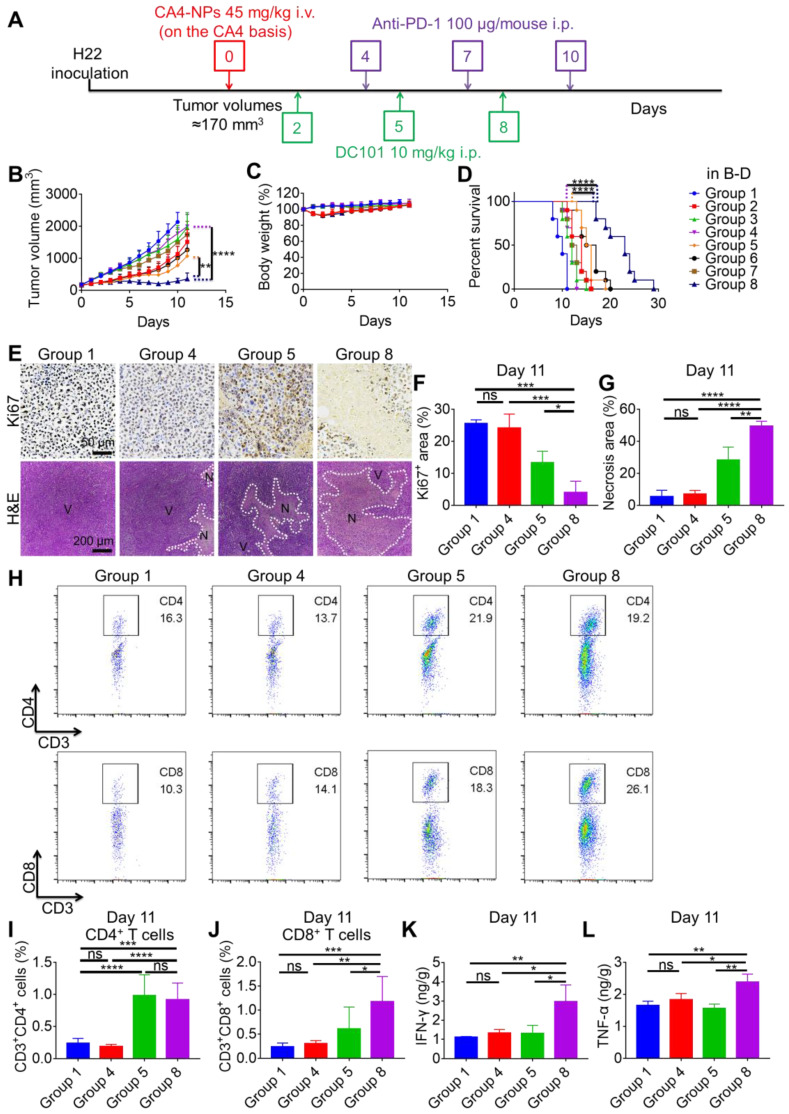
** CA4-NPs + DC101 improved the therapeutic efficacy of anti-PD-1.** (A) Schematic representation of the drug treatment strategy. (B, C, D). Tumor growth curves (B, n = 10), body weight changes (C, n = 10), and survival curves (D, n = 10) of the eight groups following drug treatment. (E) IHC staining of Ki67 and H&E for tumors in the Group 1, Group 4, Group 5, and Group 8 on day 11. V, viable region of the tumors; N, necrotic region of the tumors. (F) Quantitative analysis of the Ki67^+^ area (Ki67^+^ area/field) using the Image J software (n = 3). (G) Quantitative analysis of the necrotic area (necrotic area/field) using the Image J software (n = 3). (H) Representative flow cytometry plots for intratumoral CD4^+^ T (CD3^+^CD4^+^) cells and CD8^+^ T (CD3^+^CD8^+^) cells in the Group 1, Group 4, Group 5, and Group 8 on day 11. (I, J) Quantification of intratumoral CD4^+^ T cells (I, n = 6) and CD8^+^ T cells (J, n = 6) detected by flow cytometry. (K, L) ELISA for evaluating intratumoral IFN-γ (K, n = 3) and TNF-α (L, n = 3) levels in the Group 1, Group 4, Group 5, and Group 8 on day 11. Data are presented as mean ± SD (*P < 0.05, **P < 0.01, ***P < 0.001, ****P < 0.0001, ns, not significant). Note: PBS (Group 1); CA4-NPs (Group 2); DC101 (Group 3); anti-PD-1 (Group 4); CA4-NPs + DC101 (Group 5); CA4NPs + anti-PD-1 (Group 6); DC101 + anti-PD-1 (Group 7); and CA4-NPs + DC101 + anti-PD-1 (Group 8).

To investigate whether the combination of CA4-NPs and DC101 enhanced the therapeutic efficacy of anti-PD-1, we constructed a model of H22 tumors in Balb/c mice. When the tumor volume reached ~170 mm^3^, the mice were randomly divided into eight groups, including the following: PBS (Group 1); CA4-NPs (Group 2); DC101 (Group 3); anti-PD-1 (Group 4); CA4-NPs + DC101 (Group 5); CA4NPs + anti-PD-1 (Group 6); DC101 + anti-PD-1 (Group 7); and CA4-NPs + DC101 + anti-PD-1 (Group 8). The mice were treated as shown in Figure 4A. As shown in Figure 4B and Figure S7, the tumor volume reached ~2000 mm^3^ on day 10 in the PBS group, indicative of rapid tumor growth. The tumor volume decreased on day 2 following a single injection of CA4-NPs, suggesting that the CA4-NPs effectively reduced the tumor burden. On day 10, the tumor inhibition rates in the CA4-NP, DC101, and CA4-NP + DC101 groups were 47.0%, 25.3%, and 63.5%, respectively, suggesting that the CA4-NPs + DC101 effectively reduced the tumor burden and that the CA4-NPs primarily contributed to the reduction in tumor burden in the CA4-NP + DC101 group. On day 11, the tumor volumes were 1074.0 ± 199.7 mm^3^, 1999.2 ± 376.0 mm^3^, and 360.0 ± 188.3 mm^3^ in the CA4-NP + DC101, anti-PD-1, and CA4-NP + DC101 + anti-PD-1 groups, respectively, and the tumor volume in the CA4-NP + DC101 + anti-PD-1 group was significantly lower than that in the CA4-NP + DC101 and anti-PD-1 groups. The tumor inhibition rates on day 10 were 63.5%, 16.8%, and 86.4% in the CA4-NP + DC101, anti-PD-1, and CA4-NP + DC101 + anti-PD-1 groups, respectively, with a Q value of 1.24, which was higher than 1.15, confirming the strong synergistic effects between CA4-NPs + DC101 and anti-PD-1. As shown in Figure 4C, the body weight of mice without CA4-NP treatment showed only a negligible decrease, implying that the toxicity of the drug combination was mainly caused by CA4-NPs. The body weight of mice in the CA4-NP + DC101 + anti-PD-1 group had a maximum loss of 11.9% on day 2 of treatment, which was gradually recovered at later stages, and no mice died during the course of treatment, indicating that low systemic toxicity and side effects were induced by the triple drug combination. Next, the survival of H22-bearing mice was monitored, revealing that CA4-NP + DC101 + anti-PD-1 treatment significantly extended the survival of mice compared with all other treatments (Figure 4D). CA4-NP + DC101 + anti-PD-1 treatment significantly prolonged the median survival time (23 days), ~1.9-fold compared with anti-PD-1 treatment (12 days). Collectively, these results suggested that CA4-NPs + DC101 enhanced the efficacy of anti-PD-1 therapy in H22-bearing mice.

The tumor inhibition efficacy was further compared by IHC staining with Ki67 and H&E staining on day 11 (Figure 4E). The Ki67^+^ areas in the PBS, CA4-NP + DC101, anti-PD-1, and CA4-NP + DC101 + anti-PD-1 groups were 25.6 ± 1.2%, 13.4 ± 3.6%, 24.1 ± 4.4%, and 4.0 ± 3.5%, respectively (Figure 4F), indicating that the triple drug combination effectively inhibited tumor cell proliferation compared with the anti-PD-1 alone. The tumor inhibition efficacy was further evaluated by H&E staining, showing tumors with a diffused small area of necrosis in the anti-PD-1 group, and tumors with most areas of typical non-nuclear dead cells in the CA4-NP + DC101 + anti-PD-1 group. Quantitative analysis indicated a necrotic area of 5.5 ± 4.0%, 28.3 ± 8.1%, 7.0 ± 2.4%, and 49.4 ± 3.1% in the PBS, CA4-NP + DC101, anti-PD-1, and CA4-NP + DC101 + anti-PD-1 groups, respectively (Figure 4G), indicating that the triple drug therapy effectively induced tumor cell necrosis. Taken together, these results revealed that CA4-NPs + DC101 significantly enhanced anti-PD-1 therapy by increasing the inhibition of tumor cell proliferation and promoting tumor cell necrosis.

PD-1 is highly expressed on T cells and plays a crucial inhibitory role in T cell proliferation and CD8^+^ T cell activity via interaction with PD-L1 expressed on tumor cells 62, 63. To evaluate the synergistic effects of anti-PD-1 with CA4-NPs + DC101, intratumoral T cells were analyzed by flow cytometry on day 11. The numbers of intratumoral CD4^+^ T cells in the PBS, CA4-NP + DC101, anti-PD-1, and CA4-NP + DC101 + anti-PD-1 groups were 0.24%, 0.99%, 0.19%, and 0.92%, respectively (Figure 4H, I, and Figure S8), and the number of CD4^+^ T cells in the CA4-NP + DC101 + anti-PD-1 group was significantly higher than that in the anti-PD-1 group. However, the number of CD4^+^ T cells in the CA4-NP + DC101 + anti-PD-1 and CA4-NP + DC101 groups did not significantly differ, and the former was lower than the later, indicating that anti-PD-1 therapy failed to increase the number of CD4^+^ T cells in the CA4-NP + DC101 + anti-PD-1 group. The numbers of intratumoral CD8^+^ T cells in the PBS, CA4-NP + DC101, anti-PD-1, and CA4-NP + DC101 + anti-PD-1 groups were 0.24%, 0.61%, 0.31%, and 1.18%, respectively (Figure 4H, J, and Figure S8). Compared to the PBS group, anti-PD-1 group did not significantly increase the number of intratumoral CD8^+^ T cells. CA4-NP + DC101 + anti-PD-1 significantly increased the number of CD8^+^ T cells compared with PBS, anti-PD-1, and CA4-NP + DC101 treatment. IHC staining of CD8 confirmed that CA4-NP + DC101 + anti-PD-1 treatment resulted in an increase in the number of intratumoral CD8^+^ T cells (Figure S9). It was notable that without CA4-NPs + DC101, anti-PD-1 monotherapy did not increase the number of intratumoral CD8^+^ T cells, as no significant changes in the number of intratumoral CD8^+^ T cells were observed. In contrast, the synergizing effect of anti-PD-1 and CA4-NPs + DC101 increased the number of intratumoral CD8^+^ T cells. Previous studies have shown that CD8^+^ T cells proliferate and display recovery of function following anti-PD-1 treatment, which simultaneously requires CD4^+^ T cells to produce the cytokines for primary CD8^+^ T cell responses 64. The relative levels of proinflammatory cytokines in the tumors were therefore analyzed on day 11. Figure 4K shows that the levels of IFN-γ expression in the PBS, CA4-NP + DC101, anti-PD-1, and CA4-NP + DC101 + anti-PD-1 groups were 1.11 ± 0.04 ng/g, 1.31 ± 0.42 ng/g, 1.32 ± 0.19 ng/g, and 2.96 ± 0.87 ng/g, respectively. Figure 4L shows that the levels of TNF-α in the PBS, CA4-NP + DC101, anti-PD-1, and CA4-NP + DC101 + anti-PD-1 groups were 1.65 ± 0.14 ng/g, 1.56 ± 0.14 ng/g, 1.83 ± 0.19 ng/g, and 2.39 ± 0.25 ng/g, respectively. As IL-2 is a cytokine produced by activated T cells 65, the levels of intratumoral cytokine IL-2 after CA4-NP + DC101 + anti-PD-1 treatment were evaluate by ELISA to verify the enhancement of anti-PD-1 therapy with CA4-NPs + DC101. The levels of IL-2 expression in the PBS, CA4-NP + DC101, anti-PD-1, and CA4-NP + DC101 + anti-PD-1 groups were 0.13 ± 0.02 ng/g, 0.15 ± 0.04 ng/g, 0.19 ± 0.02 ng/g, and 0.43 ± 0.15 ng/g, respectively; further, CA4-NPs + DC101 + anti-PD-1 induced the highest IL-2 secretion compared with any other treatment (Figure S10). This supported a positive feedback for CA4-NPs + DC101 promoting anti-PD-1 therapy. In conclusion, the levels of IFN-γ, TNF-α, and IL-2 therefore significantly increased following CA4-NP + DC101 + anti-PD-1 treatment, compared with PBS and anti-PD-1 treatments. These results indicated the synergistic activity of anti-PD-1 with CA4-NP + DC101 for increasing the number of CD8^+^ T cells and activating local immune status.

Finally, the long-term safety and systemic toxicity of drugs were further investigated to assess their potential for future clinical applications. No remarkable pathological abnormalities in H&E-stained heart, liver, spleen, lung, and kidney tissues were observed on day 11 (Figure S11). These results further indicated that the CA4-NP + DC101 + anti-PD-1 therapy was well-tolerated in animal safety studies.

## Conclusion

**Scheme 1:**
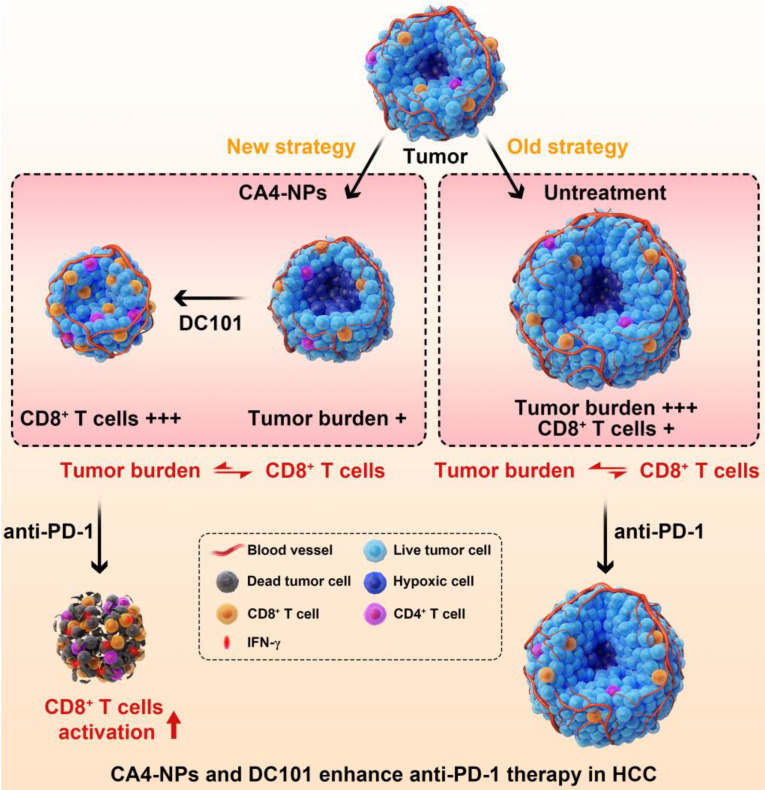
** Illustration of the combination of CA4-NPs and DC101 enhancing the therapeutic efficacy of anti-PD-1 in HCC.** 1) CA4-NPs disrupted the tumor blood vessels, leading to tumor hypoxia, and reduced tumor burden. 2) DC101 normalized the tumor vasculature, reduced tumor hypoxia, and increased the number of intratumoral CD8^+^ T cells. CA4-NPs + DC101 reduced tumor burden, while simultaneously increasing the number of intratumoral CD8^+^ T cells, and regulated the imbalance between CD8^+^ T cells and tumor burden. 3) The synergistic effects of CA4-NPs + DC101 with anti-PD-1 increased the number of intratumoral CD8^+^ T cells and increased the secretion of IFN-γ. In the absence of CA4-NPs + DC101, anti-PD-1 alone showed no significant effects with respect to the activation of CD8^+^ T cells or tumor inhibition.

In this study, to enhance the therapeutic efficacy of anti-PD-1, we established a novel concept of vascular disruption and normalization dependent on VDAs (CA4-NPs) + VEGF/VEGFR2 inhibitors (DC101) to regulate the imbalance between CD8^+^ T cells and tumor burden, following a two-step treatment regimen (Scheme 1). First, CA4-NPs disrupted the tumor blood vessels and led to tumor hypoxia and tumor cell necrosis, which effectively reduced tumor burden. Second, DC101 normalized the tumor vasculature, reduced tumor hypoxia, and increased the number of intratumoral CD8^+^ T cells. The combination of CA4-NPs and DC101, therefore, successively disrupted and normalized tumor vasculature, simultaneously reducing the tumor burden, increasing the number of intratumoral CD8^+^ T cells, and successfully regulating the imbalance between CD8^+^ T cells and tumor burden. Finally, with the regulated balance between CD8^+^ T cells and tumor burden, the number of intratumoral CD8^+^ T cells was increased and IFN-γ secretion was increased by anti-PD-1, thereby enhancing the efficacy of anti-PD-1 therapy. In the absence of CA4-NPs + DC101, anti-PD-1 alone had no significant effects on the number of intratumoral CD8^+^ T cells or tumor inhibition.

Although subcutaneous transplantation of H22 cells is one of the most widely used model in HCC, the orthotopic transplantation mouse model and human hepatoma models are more convincing for judging the pharmacodynamics of anti-tumor agents. Whether our therapeutic strategy is effective in the abovementioned models needs to be further verified.

In summary, we highlighted how regulating imbalance between CD8^+^ T cells and tumor burden with CA4-NPs + DC101 via tumor vascular disruption and normalization represented an innovative strategy to enhance the effectiveness of anti-PD-1 therapy. The effectiveness of this strategy in the clinical treatment of HCC now warrants further investigation.

## Supplementary Material

Supplementary figures and tables.

## Abbreviations

CA4: Combretastatin A4
CA4P: CA4 disodium phosphate
CA4-NPs: combretastatin A4 nanoparticles, poly (_L_-glutamic acid)-*graft*-methoxy poly (ethylene glycol)/combretastatin A4
CD31: platelet-endothelial cell adhesion molecule
DAPI: 4', 6-diamidino-2-phenylindole dihydrochloride
DLC: drug loading content
DAB: 3, 3'-diaminobenzidine
dd water: double distilled water
DMF: N, N-Dimethylformamide
ELISA: enzyme-linked immunosorbent assay
FGF: fibroblast growth factor
FBS: fetal bovine serum
HCC: hepatocellular carcinoma
H&E: Hematoxylin and eosin
HPLC: High-performance liquid chromatography
i.v.: intravenous
i.p.: intraperitoneal
IR: inhibition rate
IFN-γ: interferon-gamma
IHC: immunohistochemistry
ORR: objective response rates
OCT: optimal cutting temperature
PD-1: programmed cell death protein 1
PD-L1: programmed cell death ligand 1
PBS: phosphate-buffered saline
SD: standard deviation
TACE: transarterial chemoembolization
TEA: trimethylamine
TNF-α: tumor necrosis factor-alpha
V: volume
VEGF: vascular endothelial growth factor
VEGFR2: VEGF receptor 2
VDA: vascular disrupting agent
α-SMA: α-smooth muscle actin
