# Supplementary material for: Enhanced anti-PD-1 therapy in hepatocellular carcinoma by tumor vascular disruption and normalization dependent on combretastatin A4 nanoparticles and DC101
Source: Theranostics. 2021 Apr 3;11(12):5955–69. doi: 10.7150/thno.58164 (PMC8058708; doi:10.7150/thno.58164)
Supplement: Supplementary file 1 — Supplementary figures and tables. [file thnov11p5955s1.pdf]

Enhanced anti-PD-1 therapy in hepatocellular carcinoma by tumor vascular disruption and normalization dependent on combretastatin A4 nanoparticles and DC101

Xin Bao, Na Shen*, Yan Lou, Haiyang Yu, Yue Wang, Linlin Liu*, Zhaohui Tang*, Xuesi Chen

X. Bao, Prof. L. Liu
Department of Radiotherapy, The Second Hospital of Jilin University, Changchun 130041, P. R. China
E-mail: liulinl@jlu.edu.cn (L. Liu)
Dr. N. Shen, Dr. H. Yu, Dr. Y. Wang, Prof. Z. Tang, Prof. X. Chen
Key Laboratory of Polymer Ecomaterials, Changchun Institute of Applied Chemistry, Chinese Academy of Sciences, Changchun 130022, P. R. China
E-mail: ztang@ciac.ac.cn (Z. Tang); nshen@ciac.ac.cn (N. Shen)
X. Bao
Department of Thyroid, The Second Hospital of Jilin University, Changchun 130041, P. R. China
Y. Lou
Department of Nephropathy, The Second Hospital of Jilin University, Changchun 130041, P. R. China

Supplementary Figures

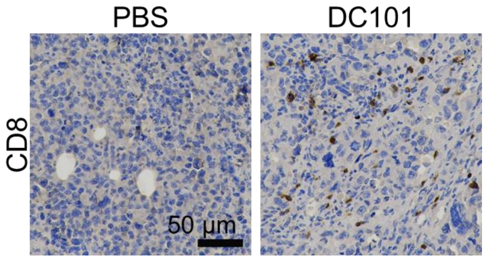

Figure S1. IHC staining of CD8 for tumors in the PBS and DC101 groups on day 11.

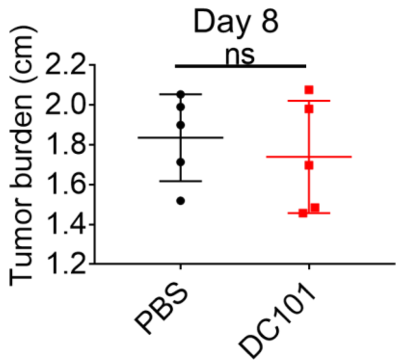

Figure S2. Tumor burden (long axis of tumors) in the PBS and DC101 groups on day 8 (n = 5). Data are presented as mean ± SD (ns, not significant).

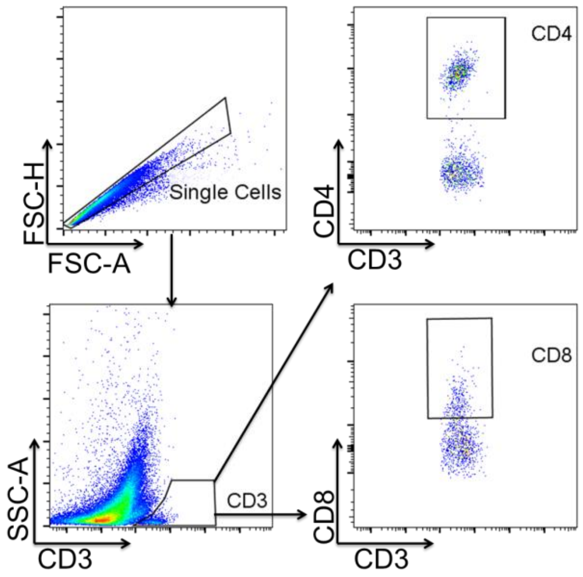

Figure S3. Gating strategies for flow cytometry detecting $CD4^+$ T cells and $CD8^+$ T cells in the PBS, CA4-NP, DC101, and CA4-NP + DC101 groups on day 10.

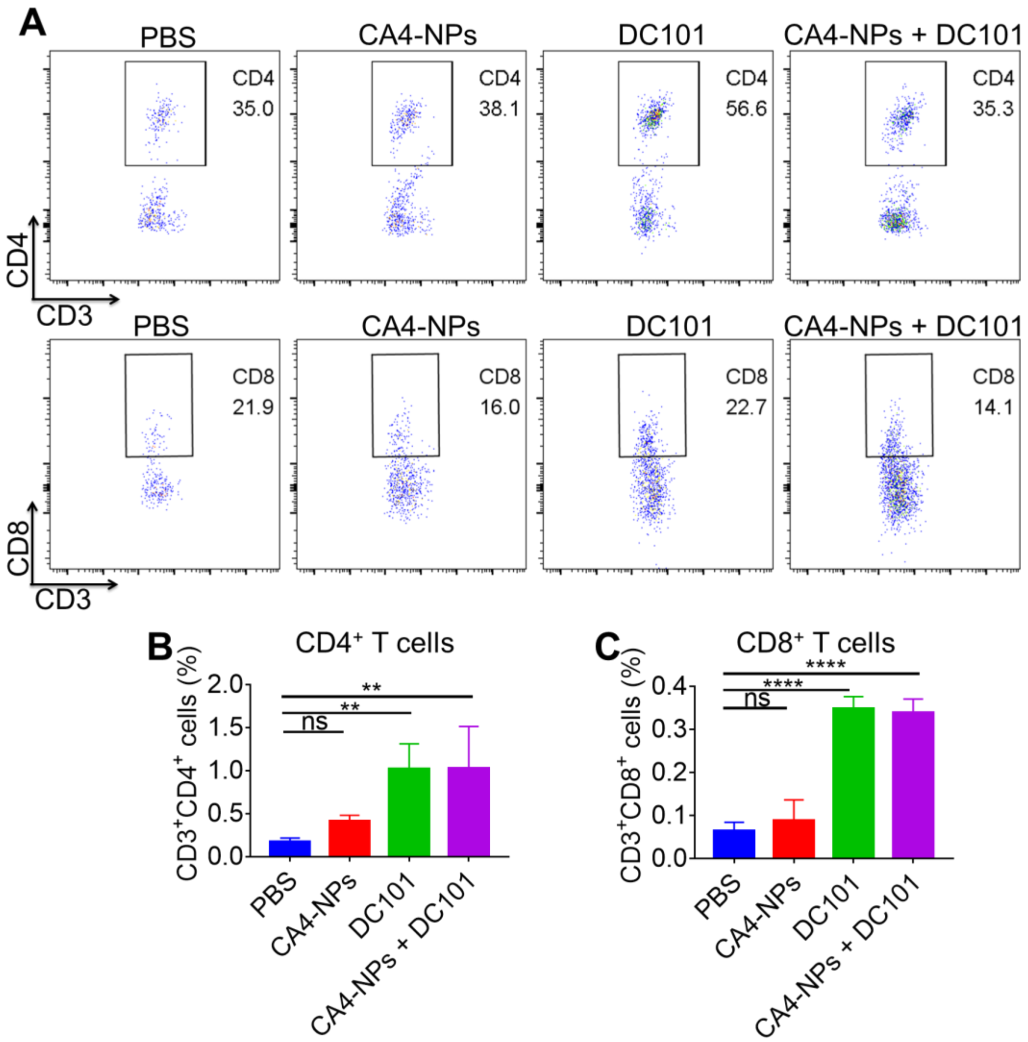

Figure S4. CA4-NPs + DC101 increased the number of intratumoral CD4$^+$ T cells and CD8$^+$ T cells. (A) Representative flow cytometry plots for intratumoral CD4$^+$ T (CD3$^+$CD4$^+$) cells and CD8$^+$ T (CD3$^+$CD8$^+$) cells in the PBS, CA4-NP + DC101, anti-PD-1, and CA4-NP + DC101 + anti-PD-1 groups on day 10. (B, C) Quantification of intratumoral CD4$^+$ T cells (B) and CD8$^+$ T cells (C) detected by flow cytometry. Data are presented as mean ± SD (***P* < 0.01, *****P* < 0.0001, ns, not significant).

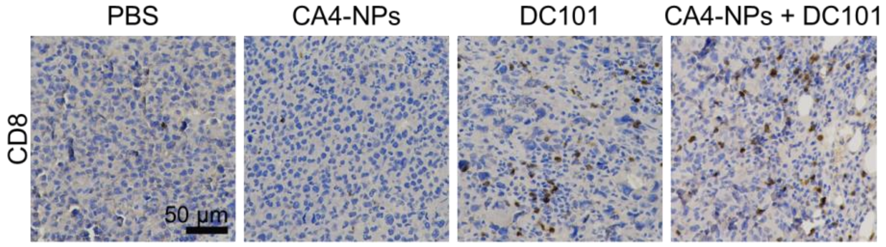

Figure S5. IHC staining of CD8 for tumors in the PBS, CA4-NP, DC101, and CA4-NP + DC101 groups on day 11.

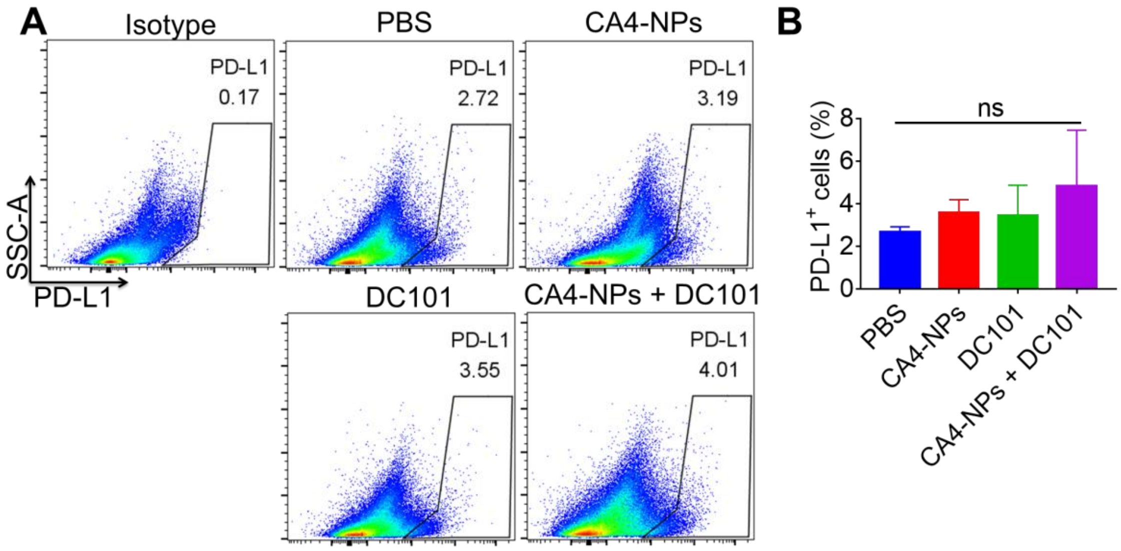

Figure S6. Expression of PD-L1 in H22 tumor after treatment. (A) Representative image for flow cytometry plots showing PD-L1 expression in tumor cells after PBS, CA4-NP, DC101, and CA4-NP + DC101 treatment on day 10. (B) Quantification of PD-L1^{+} cells detected by flow cytometry (n = 3). Data are presented as mean ± SD (ns, not significant).

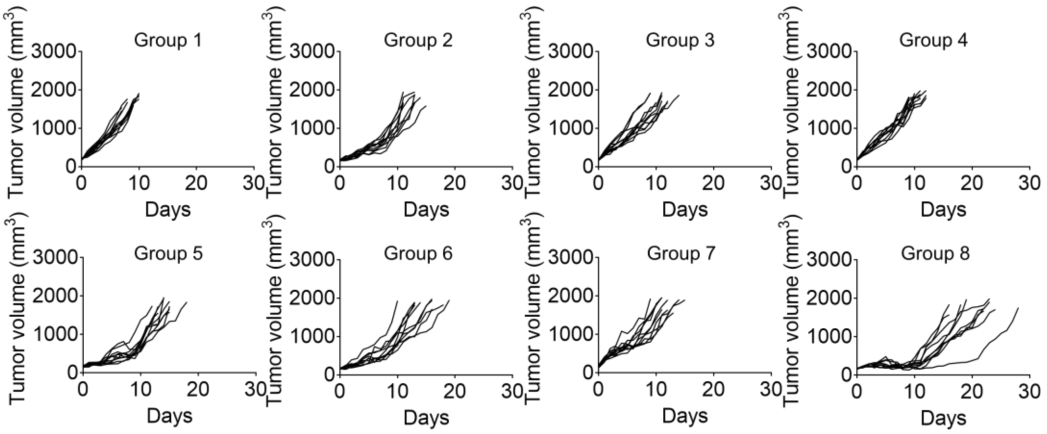

Figure S7. Tumor growth curves for individual H22-bearing mice in the eight groups (n = 10). Note: PBS (Group 1); CA4-NPs (Group 2); DC101 (Group 3); anti-PD-1 (Group 4); CA4-NPs + DC101 (Group 5); CA4NPs + anti-PD-1 (Group 6); DC101 + anti-PD-1 (Group 7); and CA4-NPs + DC101 + anti-PD-1 (Group 8).

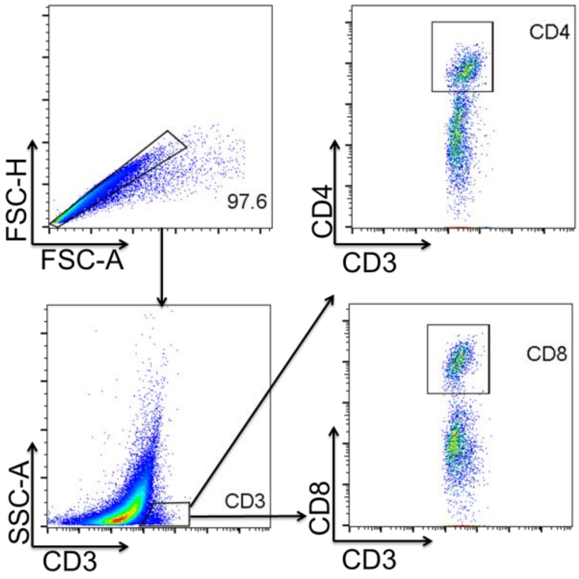

Figure S8. Gating strategies for flow cytometry detecting CD4$^+$ T cells and CD8$^+$ T cells in the PBS, CA4-NP + DC101, anti-PD-1, and CA4-NP + DC101 + anti-PD-1 groups on day 11.

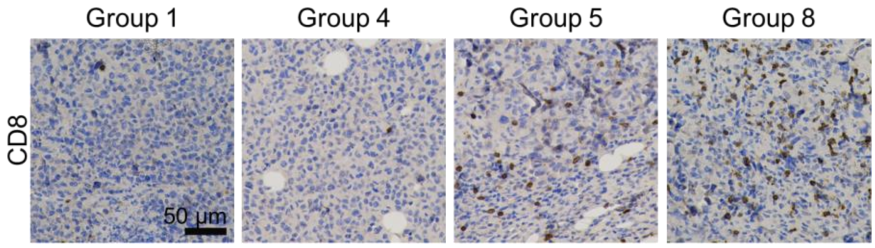

Figure S9. IHC staining of CD8 for tumors in the Group 1, Group 4, Group 5, and Group 8 on day 11. Note: PBS (Group 1); anti-PD-1 (Group 4); CA4-NPs + DC101 (Group 5); and CA4-NPs + DC101 + anti-PD-1 (Group 8).

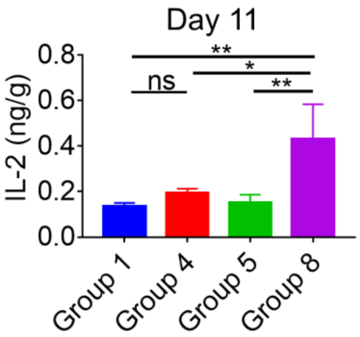

Figure S10. ELISA results evaluating intratumoral IL-2 levels in the Group 1, Group 4, Group 5, and Group 8 on day 11 (n = 3). Data are presented as mean ± SD (**P* < 0.05, ***P* < 0.01, ns, not significant). Note: PBS (Group 1); anti-PD-1 (Group 4); CA4-NPs + DC101 (Group 5); and CA4-NPs + DC101 + anti-PD-1 (Group 8).

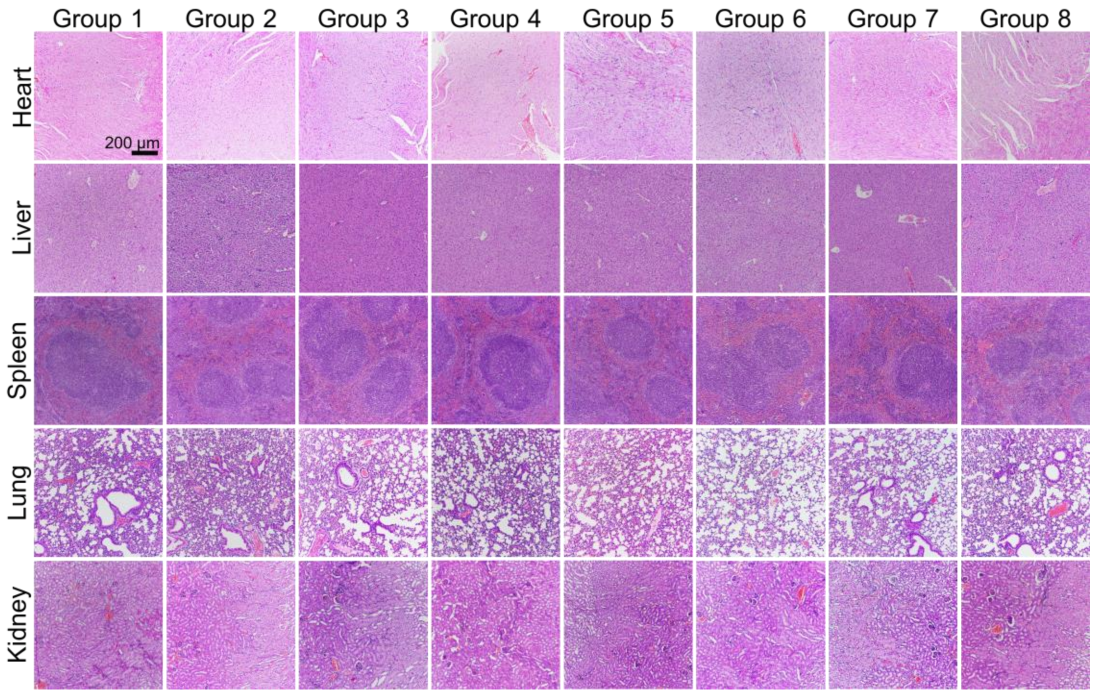

Figure S11. H&E staining of the heart, liver, spleen, lung, and kidney tissues on day 11 in the eight groups. Note: PBS (Group 1); CA4-NPs (Group 2); DC101 (Group 3); anti-PD-1 (Group 4); CA4-NPs + DC101 (Group 5); CA4NPs + anti-PD-1 (Group 6); DC101 + anti-PD-1 (Group 7); and CA4-NPs + DC101 + anti-PD-1 (Group 8).

Supplementary Tables

Table S1. List of antibodies used in the experiments.

Antibodies	Company	Catalog	Application
**DC101**	BioXCell	BE0060	Treatment
**Anti-PD-1**	BioXCell	BE0146	Treatment
**FITC anti-mouse CD3 Antibody**	BioLegend	100204	Flow cyt
**PE/Cy7 anti-mouse CD4 Antibody**	Invitrogen	25-0042-82	Flow cyt
**PE anti-mouse CD4 Antibody**	Invitrogen	12-0041-82	Flow cyt
**APC anti-mouse CD8a Antibody**	BioLegend	100712	Flow cyt
**PE anti-mouse PD-L1 Antibody**	Invitrogen	12-5982-82	Flow cyt
**Ki67 Rabbit Antibody**	Servicebio	GB13030-2	IHC
**Anti-CD31 Antibody**	Abcam	ab182981	IHC
**Anti-CD8 Rabbit Antibody**	Servicebio	GB13429	IHC
**HPR Goat anti-Rabbit IgG**	Servicebio	GB23303	IHC
**APC anti-mouse CD31 Antibody**	eBioscience	17-0311-80	IF
**Anti-α-SMA Antibody**	Abcam	ab7817	IF
**Cy3 Goat anti-mouse IgG**	ABclonal	AS008	IF

Flow cyt, Flow cytometry; IF, Immunofluorescence; IHC, Immunohistochemistry

Table S2. Nuclear dye used in the experiments.

Dye	Company	Catalog
**DAPI**	Sigma	D9542
**SYTOX Green Nucleic Acid Stain**	Thermo Fisher	S7020

DAPI, 4', 6-diamidino-2-phenylindole dihydrochloride

Table S3. ELISA kits used in the experiments.

ELISA kits	Company	Catalog
**Mouse-IFNgamma ELISA kit**	Anoric	TAE-366m
**Mouse-TNFalpha ELISA kit**	Anoric	TAE-569m
**IL-2 Mouse Uncoated ELISA Kit**	Thermo Fisher	88-7024-88
**Mouse-VEGF ELISA kit**	Lengton	BPE20260

ELISA, Enzyme-linked immunosorbent assay; IFN, Interferon; TNF, Tumor necrosis factor; VEGF, Vascular endothelial growth factor
